# Radiofrequency Thermoablation On *Ex Vivo* Animal Tissues: Changes on Isolated Swine Thyroids

**DOI:** 10.3389/fendo.2021.575565

**Published:** 2021-06-10

**Authors:** Paola Pregel, Elisa Scala, Michela Bullone, Marina Martano, Linda Nozza, Sara Garberoglio, Roberto Garberoglio, Enrico Bollo, Frine Eleonora Scaglione

**Affiliations:** ^1^ Dipartimento di Scienze Veterinarie, Università degli Studi di Torino, Torino, Italy; ^2^ Evidensia Specialisthästsjukhuset, Strömsholm, Sweden; ^3^ Dipartimento di Scienze Medico-Veterinarie, Università di Parma, Parma, Italy; ^4^ Dipartimento di Scienze Mediche, Università degli Studi di Torino, Torino, Italy

**Keywords:** RFA, thyroid, swine, perfused electrodes, hypertonic saline

## Abstract

The use of Radiofrequency thermoablation (RFA) for treating large thyroid nodules is limited by the modest efficiency of the available systems in terms of volume of the ablation zones (AZs). This increases the risk of incomplete ablation of the nodule. Systems employing perfused electrodes have been developed to increase the volume of the AZ. Aim of this study is to compare the size of the AZ induced by RFA systems using internally cooled perfused vs. non-perfused electrodes in swine thyroids. RFAs were performed on 40 freshly isolated swine thyroids using both systems. The perfused system was tested using 0.9% saline, 7% and 18% hypertonic saline solutions. Energy delivery to the tissue was stopped when tissue conductivity dropped (real life simulations) and after an established time of 20 seconds (controlled duration). Following RFA, thyroids were transversally and longitudinally cut. Photographs were taken for macroscopic morphometry of the ablated zones before and after formalin fixation, to evaluate tissue shrinkage. Microscopic morphometry was performed on PAS stained sections. In real life simulation experiments, gross morphometry revealed that AZs produced with electrodes perfused using 7.0% saline are larger compared to isotonic saline. Microscopically, all the conditions tested using the perfused system produced larger AZs compared to non-perfused system after 20 seconds of RFA. In conclusion, the perfusion with 7.0% NaCl solution increased the electrical conductivity of the tissue in real life simulations, resulting in larger ablated areas compared to the use of isotonic saline.

## Introduction

In the last thirty years, minimally invasive techniques have been consistently raising in importance in human medicine. Radiofrequency ablation (RFA) is the most extensively studied and widely applied technique in human clinical practice (1), largely used to treat malignant and benign tumors in several organs (1, 2). It consists on the direct placement of one or more radiofrequency (RF) electrodes into the tumor tissue under ultrasound (US), computed tomography (CT) or magnetic resonance (MR) guidance (2). RF electrodes apply thermal energy (hyperthermia) through the passage of high-frequency alternating current through the electrode, producing tissue necrosis of the tumor, without the need for surgical removal. Among other organs, the application of RFA has been extended in the last decade to the treatment of thyroid neoplasia and nodular thyroid disease, with a proven efficacy in terms of reduction of thyroid nodule mean volume and improvement of the patient symptoms (3–7). To date, RFA is considered a safe alternative to surgery for benign thyroid nodules, and it is proposed for treatment of primary thyroid carcinomas and recurrent thyroid cancer (8–16).

Nodular thyroid disease refers to the presence of abnormal masses in the thyroid gland. It is a very common finding in clinical practice, with a good prognosis (17–19). Thyroid nodules are most frequently asymptomatic, stable or slow-growing over time, and require no treatment. Clinicians’ concerns in these cases are imputable mainly to the space-occupying effect of such lesions, as well as to the associated endocrine disorders, such as hyperthyroidism and hypothyroidism. Large thyroid nodules may cause compression to the surrounding structures, neck discomfort, cosmetic complaints, and decreased quality of life. To date, the possible treatments include surgery, radioiodine therapy, TSH-suppression therapy, ethanol injection, and hyperthermic methods (18). The main limit of the latter is the small size of the induced AZ, considered adequate for treating lesions up to 5 cm^2^ (2).

Tissue perfusion with saline has been shown to improve the performances of RFA systems in terms of volume of the AZs (20). The aim of this study was to compare the performances of RFA systems employing internally cooled and perfused vs. internally cooled and non-perfused electrodes in terms of AZ size, using ex-vivo swine thyroids. Moreover, we sought to determine whether using solution with increased osmolarity produced any effect on the size of AZs. Anatomic and physiologic characteristics of swine make them useful models in certain areas of surgical research (21). In particular, swine thyroid glands have an adequate size and a fine tissue structure resembling those of humans.

## Materials and Methods

### Thermal Ablation Procedures

Forty freshly isolated swine thyroids from a pig slaughterhouse were stored at refrigerated temperature until RFA exposure, and then ablated using the fixed-shot technique. The moving shot technique, usually applied in thyroid treatment, was not evaluated in this work, since results are dependent on the number of passages and the tilt angles of the electrode. The single passage allowed a better measurement of the parameters obtained by a single ablation treatment.

Radiofrequency ablation procedures were performed using either a perfused or non-perfused system, with internally cooled and perfused (RFTS 1010N, RF Medical Co. Ltd., Korea) or internally cooled and non-perfused electrodes (RFT 1010N, RF Medical Co. Ltd., Korea), respectively. A high frequency alternating current generator specific for radiofrequency thermal ablation (RF Generator M-3004, RF Medical Co. Ltd., Korea), equipped with a peristaltic pump, was used to power the system.

A starting power output of 80 Watt was used. Ground pads were placed under each organ to close the RT circuit, placing a swine liver between the ground pads and the thyroid, to ensure an adequate distance and obtain a correct electrical conduction. Procedures were stopped using an impedance-based system. All electrodes used were monopolar, 10 cm in length, and 18G in diameter, with an exposed electrode of 10 mm. Internally cooled and non-perfused (IC) electrodes are provided with an internal cooling system pumping cold (5°C) sterile saline solution. This prevents the electrodes to reach temperatures above 100°C, which could induce tissue carbonization. Internally cooled and perfused (wet tip, WT) electrodes have a second system for instillation of liquids into the tissues while RFA is performed by means of a peristaltic pump. Cold (5°C) sterile isotonic solution 0.9% NaCl, hypertonic 7.0% NaCl solution, and hypertonic 18.0% NaCl solution were used in our study to perfuse thyroid parenchyma.

Energy delivery to the tissue was stopped when tissue conductivity dropped (real life simulations) and after an established time of 20 seconds (controlled duration).

### Morphometric Analysis

The criteria proposed by Mulier and colleagues (22) were adopted for standardized description of the AZ size. At the end of the ablation procedure, the RF electrode was left *in situ* in order to easily identify the treated area. The organ was cut along the shaft of the RF electrode (axial plane of the AZ) obtaining the two orthogonal planes of the lesion. Then both halves of the AZ were cut in the transverse plane, perpendicular to the electrode, at the site of the largest transverse diameter of the coagulation zone.

Morphometric analysis of the macroscopically visible AZs was performed with the software *ImageJ 1.51j8* (National Institutes of Health, USA) on pictures taken from both faces of each plane (axial and transversal), including a calibrator in each picture. Thyroids were then individually fixed in buffered formalin (10%) and pictures and measurements of the parameters to assess the degree of tissue shrinkage were taken a second time. Area and perimeter of the AZ were calculated for each image. Both faces of each plane were measured, and mean data used for analyses. The volume of the rotation solid (lesion volume, calculated as: $Volume=\frac{4}{3}\pi \;abc$, where a, b and c were halves of each axis, assuming that it is ascribable to the area of a rotation (23) ellipsoid) and the equivalent diameter (diameter that a round section would have with the same proportion of Perimeter P and Area A) were estimated from the former parameters. The semiaxes of the lesion were also measured.

Microscopic morphometry of the AZs was performed using an Olympus BX40 microscope and the software NIS-Elements F 2.30 (Nikon Corporation, Japan) on Periodic Acid Schiff (PAS) stained sections (20x magnification), following paraffin-embedding and 4µm cut. The parameters measures were the same already described for macroscopic morphometry. A 20x magnification was selected, allowing a general view of the lesion area, taking multiple pictures of the same slide when necessary, to include the whole lesion. Pictures were assembled with the software Adobe Photoshop CC 2018 (Adobe Systems Incorporated, USA).

### Statistical Analysis

The statistical analysis of the morphometric values was performed using the software GraphPad Prism (vers. 6; GraphPad Software, California, USA). The Kolmogorov and Smirnov test was used to analyze data distribution. For the morphometric measurements, when the effect of a single variable was assessed among multiple groups, one-way ANOVA test was used, followed by Dunnett’s post-tests, if the distribution was parametric; otherwise the Kruskal-Wallis test and Dunn’s post-tests were employed. When the effect/interaction of two variables was evaluated among multiple groups, the two-way ANOVA test was used, followed by Tukey post-tests, with the Sidak correction. Paired Student’s T-test was used for the comparison between lesions on unfixed and formalin-fixed (FF), whereas the non-parametric Wilcoxon test was applied for the comparison between the two semi-axis of the rotation solid on unfixed thyroids. In order to compare differences in measurements between the same lesion parameters analyzed on gross unfixed thyroids images and on the corresponding microphotographs, Paired Student’s T-test or the non-parametric homologous Wilcoxon test were used. The coefficient of variation was evaluated to highlight the difference between repeated measurements, such as Area and Equivalent Diameter, available only in gross morphometric analysis, for both longitudinal and transverse views. All the results with a p-value<0.05 were considered statistically significant.

## Results

### Macroscopic Investigations on Unfixed Thyroids

**Figure 1:**
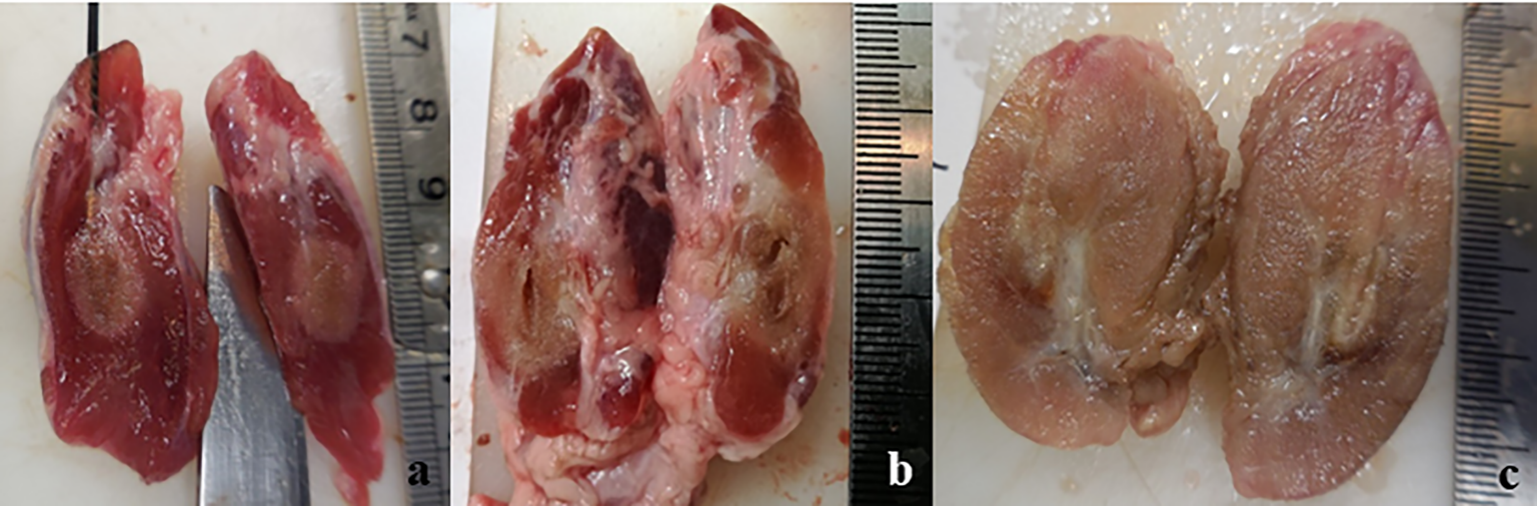
Longitudinal section of unfixed thyroids: **(A)** treated with IC electrode, 20 seconds fixed time of treatment; **(B)** treated with WT electrode, 0.9% NaCl solution, 20 seconds fixed time of treatment; **(C)** treated with WT electrode, 7.0% NaCl solution, variable time of treatment (4 minutes and 30 seconds).

The macroscopic investigation of the thyroids treated with RFA revealed an area clearly identifiable from the surrounding, normal tissue. The area could be roughly described as an ovoid, even though it was often irregular. In the unfixed thyroids, the ablated area was divided in a brown centre, in which the tissue was in direct contact with the electrode, surrounded by a pale pink area. Minimal or non-existent hemorrhagic transitional zone between ablation and normal tissue was noticed. This partition was evident in some samples more than in others, and the type of applicator seemed to slightly influence it. In fact, when the IC electrode was used, the partition was evident, and the ablation had a definite outline (**Figure 1A**). In the case of WT electrode, independently from the solution used, the outline of the lesion appeared vanishing (**Figure 1B**). The thyroid was entirely or almost interested by the coagulation area in case a WT was used, especially when 7.0% NaCl solution was injected (**Figure 1C**).

In thyroids treated with IC electrode, the treatment was fixed at 20 seconds. Regarding WT electrodes, when applying a variable time (real life simulations), RF electrode was left to work until the impedance was enough high to automatically turn the generator off.

As a result, some organs were totally thermally ablated before the generator turned off, so that it had to be manually interrupted. In these groups of organs, the potential of WT electrode was evident, but the size of the lesion was strongly influenced by the size and margins of the thyroid. In order to determine the effects of saline solution on ablation size, a procedure at fixed time was also implemented. In this case, the duration of treatment was fixed at 20 seconds, forcing the generator to stop, independently from the completion of ablation. The latter procedure was implemented due to the small size of thyroids and following the finding that many of them were completely necrotized, thus interfering with a precise measurement of the ablation (being the size of the lesion strongly dependent from the margins and size of the thyroid). This procedure was not meant to be applied in clinic but to better understand the potential of saline use.

#### Macroscopic Morphometric Data From Unfixed Thyroid

The morphometric analysis data obtained from unfixed thyroids are stated in **Table 1**, reporting mean values and standard deviation for each group.

**Table 1:** Unfixed thyroids morphometric data.

Electrode	Solution	Time of treatment	Transverse section		Longitudinal section		a (mm)	b (mm)	c (mm)	Volume (mm³)
			Area (mm²)	Equivalent diameter (mm)	Area (mm²)	Equivalent diameter (mm)				
Internally cooled	–	20-sec fixed time	50.5 ± 8.9	7.4 ± 0.5	88.1 ± 19.7	8.6 ± 1.7	3.9 ± 0.6	7.2 ± 1.1	3.8 ± 0.3	448.6 ± 121.8
Wet tip	0.9% NaCl	Variable time	142.8 ± 76.4	11.6 ± 2.3	300.2 ± 169.6	15.3 ± 4.7	6.9 ± 2.4	13.2 ± 4.3	6.1 ± 1.4	2665.0 ± 1997.0
Wet tip	7.0% NaCl	Variable time	306.8 ± 41.7	17.3 ± 1.6	699.7 ± 112.6	23.7 ± 3.0	10.7 ± 1.0	20.8 ± 1.6	9.5 ± 0.7	8916.0 ± 1660.0
Wet tip	0.9% NaCl	20-sec fixed time	82.8 ± 22.6	9.0 ± 1.3	99.3 ± 38.6	9.7 ± 2.2	4.4 ± 1.2	7.1 ± 1.2	5.1 ± 0.9	704.4 ± 368.8
Wet tip	7.0% NaCl	20-sec fixed time	70.9 ± 28.7	8.1 ± 2.0	109.7 ± 35.7	9.7 ± 1.2	4.4 ± 0.7	7.9 ± 1.6	4.1 ± 1.1	620.5 ± 317.3
Wet tip	18.0% NaCl	20-sec fixed time	71.2 ± 12.3	7.7 ± 0.6	137.0 ± 65.4	11.3 ± 3.3	4.6 ± 1.5	9.1 ± 1.7	5.3 ± 0.8	1006 ± 551.7

Overview of the mean values of Area and Equivalent diameter for both transverse and longitudinal sections, a, b, and c semiaxes, and lesion volume.

#### Effects of Time of Treatment and Solution on WT Electrode Performances

The performance of WT electrode on unfixed thyroids was analyzed through a comparison based on the used solution (0.9% or 7.0% NaCl) and the duration of treatment (variable or 20 seconds/fixed time).

**Figure 2:**
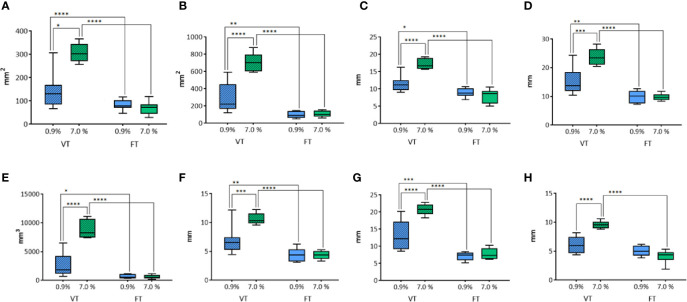
Comparison of different parameters among the groups treated with WT, with variable and fixed time of treatment, and 0.9% and 7.0% NaCl solution on thyroids. In details, **(A)** transverse area; **(B)** longitudinal area; **(C)** transverse equivalent diameter; **(D)** longitudinal equivalent diameter; **(E)** volume; **(F)** semi-axis “a”; **(G)** semi-axis “b”; **(H)** semi-axis “c”. VT, Variable Time treatment; FT, Fixed Time treatment; 0.9%= solution perfused into the tissue at 0.9% NaCl; 7.0%= solution perfused into the tissue at 7.0% NaCl; *P < 0.05; **P < 0.01; ***P < 0.001; ****P < 0.0001.

The transverse area was influenced by both the solution used (p<0.0004) and the duration of treatment (p<0.0001) (**Figure 2A**). In the groups where the time of treatment was variable, a significant increase of ablation area was revealed when the 7% solution was used in comparison to 0.9% NaCl solution (p<0.05). No difference was observed in fixed time groups (p=0.8694). The groups treated for a fixed time both with 0.9% and 7% NaCl solution showed a significant reduction of ablation area in comparison to the groups treated with the same solutions for a variable time (p<0.0001).

In addition, also the longitudinal area was influenced by the solution (p<0.0001) and the time of treatment (p<0.0001) (**Figure 2B**). As for the transverse area, the groups treated for a variable time enlightened a significantly greater area of ablation with 7.0% NaCl solution (p<0.0001). No significant difference was observed in fixed time groups (p=0.9782). In the groups treated for a fixed time the area of ablation was significantly smaller than the corresponding groups treated for a variable time (respectively, p<0.01 for 0.9% NaCl and p<0.0001 for 7.0% NaCl).

The same findings were obtained for the other parameters evaluated. The transverse equivalent diameter (**Figure 2C**) was influenced both by the solution (p=0.003) and the time of treatment (p<0.0001). Within Variable Time treatment (VT) groups, a significantly longer equivalent diameter was measured with 7.0% NaCl solution (p<0.0001). No significant difference was observed in fixed time groups (p=0.6008). In the groups treated with 0.9% NaCl solution, a significantly shorter equivalent diameter was detected for Fixed Time treatment (FT) in comparison to the corresponding VT group (respectively, p<0.05 for 0.9% NaCl, p<0.0001 for 7.0% NaCl treated groups).

The longitudinal equivalent diameter (**Figure 2D**) was influenced by the solution (p=0.0016) and the duration of treatment (p<0.0001), too. In the VT groups a significantly longer equivalent diameter was reported along with the use of 7.0% NaCl solution (p=0.0001). No difference was revealed in fixed time groups (p=0.9994). In the two VT groups a longer equivalent diameter was calculated when compared to the corresponding fixed time groups (respectively, p<0.01 for 0.9% NaCl and p<0.0001 for 7.0% NaCl).

The volume (**Figure 2E**) was influenced by the solution (p<0.0001) and the duration of treatment (p<0.0001). In the groups where the time of treatment was variable, a significant difference according to the use of 0.9% or 7.0% NaCl solutions (p<0.0001) was detected. No difference was observed in fixed time groups (p=0.9903). In the groups treated with 0.9% NaCl solution, a significant difference between variable and fixed time (p<0.05) was observed, as for the groups treated with 7.0% NaCl (p<0.0001).

The semiaxis “a” (**Figure 2F**) was influenced by the solution (p=0.0036) and the time of treatment (p<0.0001). In the groups where the time of treatment was variable, a significant increase of volume was detected according to the use of 7.0% NaCl solution when compared to 0.9%NaCl (p=0.0005). No difference was revealed in fixed time groups (p=0. 9995). In the groups treated with 0.9% NaCl solution, a significant difference between variable and fixed time (p<0.01) was detected, as for the groups treated with 7.0% NaCl (p<0.0001).

Similarly, the semiaxis “b” (**Figure 2G**) was influenced by the solution (p=0.0003) and the time of treatment (p<0.0001). Again, in VT groups a significant increase of semiaxis b length was detected when 7.0% NaCl solution was used instead of 0.9% (p<0.0001). No difference was observed in fixed time groups (p=0. 8206). In both the VT groups the semiaxis “b” was longer than in the corresponding FT groups (p<0.001 for 0.9% and p<0.0001 for the 7.0% NaCl, respectively).

The semiaxis “c” (**Figure 2H**) was influenced by the solution (p=0.0091) and the time of treatment (p<0.0001). In the groups where the time of treatment was variable, a significant difference related to the use of 0.9% or 7.0% NaCl solutions (p<0.0001) was detected. No difference was revealed in fixed time groups (p=0.1704). The time of treatment influenced the semiaxis “c” only in the groups treated with 7.0% NaCl solution (p<0.0001). No significant difference between variable and fixed time was found in the groups treated with 0.9% NaCl.

#### Comparison of IC and WT Electrodes Performances

The performance of WT electrodes (with 0.9%, 7.0% and 18.0% NaCl) was compared to IC electrode (control group) at fixed duration of treatment of 20 seconds.

**Figure 3:**
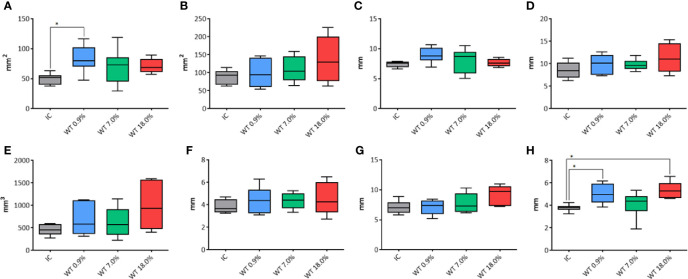
Comparison of different parameters among the groups treated with IC electrode and WT electrode with different saline solutions on unfixed thyroids. In details, **(A)** transverse area; **(B)** longitudinal area; **(C)** transverse equivalent diameter; **(D)** longitudinal equivalent diameter; **(E)** volume; **(F)** semi-axis “a”; **(G)** semi-axis “b”; **(H)** semi-axis “c”. IC, Internally cooled tip; WT, Internally cooled wet tip; WT 0.9%-7.0%-18.0%= solutions at 0.9%-7.0%-18.0% NaCl perfused into the tissue by the WT electrode; *P < 0.05.

For the transverse area (**Figure 3A**) a significant difference was shown among groups (p=0.0339) (in particular, between the control group IC and WT using 0.9% NaCl solution, p=0.014). No differences were found between the control group IC and other WT groups. For the longitudinal area no differences were observed using IC or WT electrodes (p=0.232) at 20 seconds fixed time treatment (**Figure 3B**). The transverse equivalent diameter (**Figure 3C**) and the longitudinal equivalent diameter (**Figure 3D**) were not affected by the use of either IC or WT electrodes at 20 seconds fixed time treatment (p=0.0991 and p=0.2239, respectively). The volume (**Figure 3E**) was not affected by the use of either IC or WT electrodes (p=0.2669) at 20 seconds fixed time treatment. The semiaxis “a” was not affected by the use of either IC or WT electrodes (p=0.6584) at 20 seconds fixed time treatment (**Figure 3F**). The semiaxis “b” was found not to be affected by the use of either IC or WT electrodes (p=0.0797) at 20 seconds fixed time treatment (**Figure 3G**). Regarding the semiaxis “c” (**Figure 3H**), a significant difference among groups was shown (p=0.0071). In particular, a significant longer measure was noticed in WT 0.9% NaCl solution (p=0.0215) and in WT 18.0% NaCl (p=0.0122) in comparison to IC control group. No differences were found between the control group IC and WT 7.0% (p=0.8493).

#### Comparison of the Semiaxes of the Rotation Solid

The two semi-axes of the rotation solid “a” and “c” are considered equivalent.

**Figure 4:**
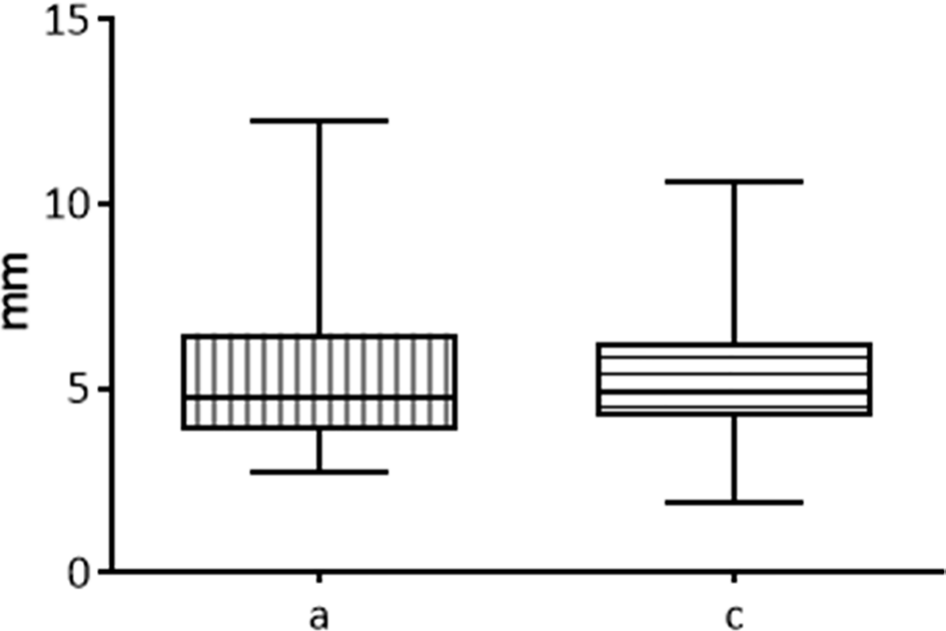
Comparison between the two semi-axis “a” and “c” of ablated area in fresh thyroids, 20 seconds fixed time of treatment.

In the comparison between the two semi-axes of the rotation solid “a” and “c” of all the unfixed thyroids belonging to fixed time groups no significant difference was detected (p=0.4939) (**Figure 4**).

### Macroscopic Investigations on Formalin-Fixed Thyroids

**Figure 5:**
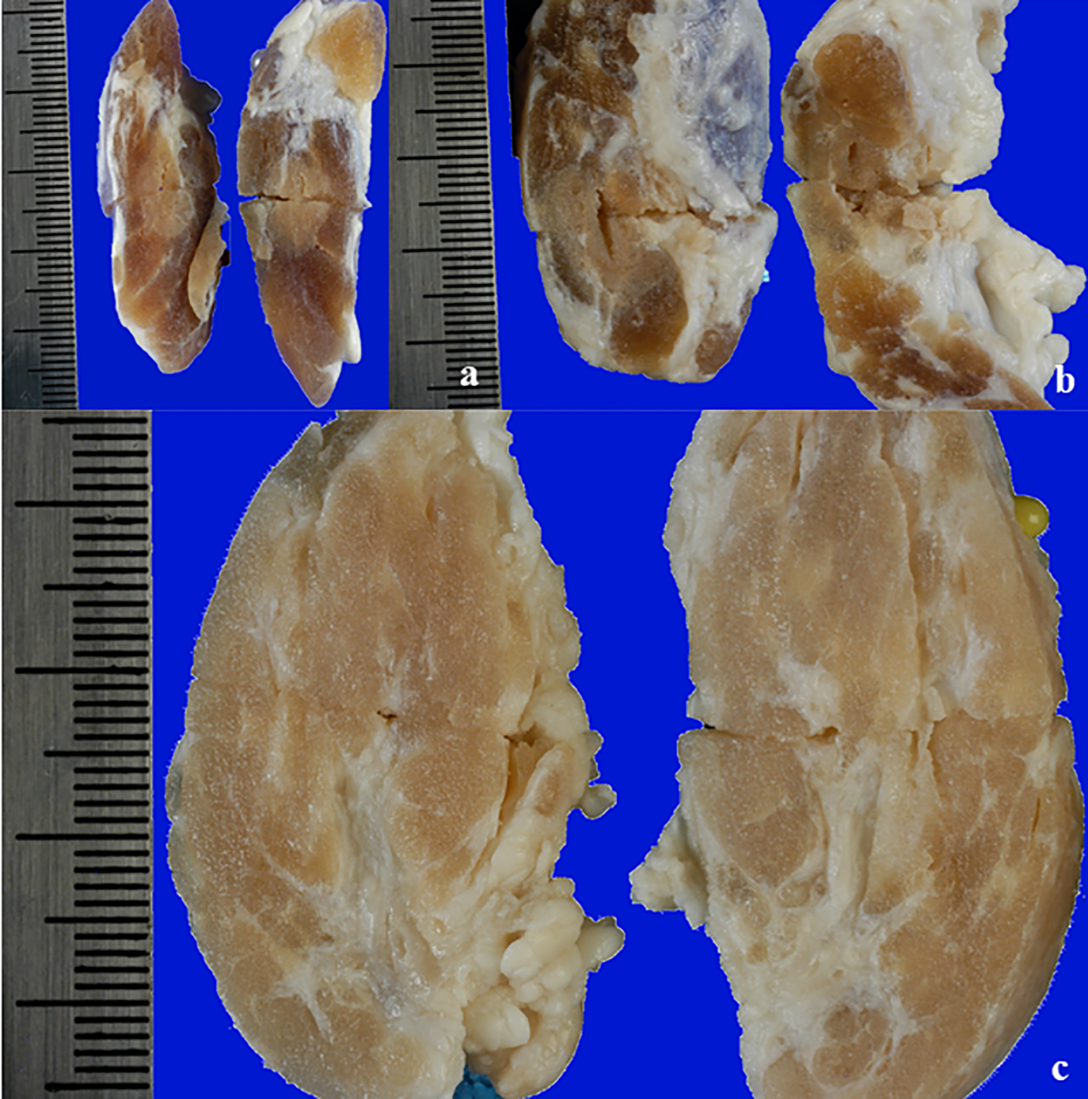
View of a longitudinal section of a FF thyroid: **(A)** treated with IC electrode, 20 seconds fixed time of treatment; **(B)** treated with WT electrode, 0.9% NaCl solution, 20 seconds fixed time of treatment; **(C)** treated with WT electrode, 7.0% NaCl solution, variable time of treatment (4 minutes and 30 seconds).

The ablated tissue was clearly detectable in FF thyroids as a pale, yellowish area, with no evident internal subdivision. Sometimes the lesion margins were more clearly evident compared to unfixed organs, even though the outline of the lesion in the thyroids treated with WT electrode was still vanishing (**Figure 5**).

### Macroscopic Morphometric Data From Formalin-Fixed Thyroids

The morphometric analysis data reporting mean values and standard deviation for each group, obtained from FF thyroids are conveyed in **Table 2**.

**Table 2:** FF thyroids morphometric data.

Electrode	Solution	Time of treatment	Transverse section		Longitudinal section		a (mm)	b (mm)	c (mm)	Volume (mm³)
			Area (mm²)	Equivalent diameter (mm)	Area (mm²)	Equivalent diameter (mm)				
Internally cooled	–	20-sec fixed time	51.2 ± 8.3	7.8 ± 0.6	69.4 ± 21.6	7.5 ± 2.1	3.4 ± 0.8	6.2 ± 0.9	3.7 ± 0.6	351.6 ± 146.1
Wet tip	0.9% NaCl	Variable time	108.2 ± 50.7	10.4 ± 1.5	133.6 ± 29.5	10.3 ± 1.1	4.7 ± 0.6	9.0 ± 2.1	5.4 ± 1.7	1010.9 ± 536.8
Wet tip	7% NaCl	Variable time	295.6 ± 7.4	14.9 ± 0.6	540.4 ± 160.2	20.1 ± 4.5	9.7 ± 0.9	17.7± 3.5	9.3 ± 0.9	6777.0 ± 2585.5
Wet tip	0.9% NaCl	20-sec fixed time	89.6 ± 23.1	9.3 ± 1.8	108.1 ± 53.1	9.9 ± 3.0	4.5 ± 1.4	7.3 ± 1.4	4.9 ± 0.9	754.8 ± 525.4
Wet tip	7% NaCl	20-sec fixed time	82.2 ± 21.5	9.2 ± 1.2	133.3 ± 42.5	10.5 ± 0.4	4.9 ± 0.5	8.6 ± 1.8	4.4 ± 0.7	793.1 ± 365.9
Wet tip	18% NaCl	20-sec fixed time	96.9	9.0	135.1	13.7	4.5	9.6	5.4	969.0

Overview of the mean values of Area and Equivalent diameter for both transverse and longitudinal sections, a, b, and c semiaxes, and lesion volume.

### Comparison Between Unfixed and Formalin-Fixed Thyroids

The pictures of unfixed thyroids were compared to the corresponding ones of formalin-fixed thyroids. Only fixed time groups were considered to lower the variability.

The compared measurements analyzed are reported in **Table 3**.

**Table 3:** Morphometric data of macroscopic images from unfixed thyroid and corresponding formalin-fixed ones.

Measurement	Unfixed thyroids		FF thyroids	
	VT+FT	FT	VT+FT	FT
Transverse area (mm²)	100.0 ± 71.7	70.22 ± 25.5	102.0 ± 72.7	72.8 ± 24.2
Longitudinal area (mm²)	178.4 ± 191.5	102.1 ± 36.3	145.5 ± 138.6	96.9 ± 43.2
Transverse equivalent diameter (mm)	9.7 ± 3.0	8.3 ± 1.1	9.6 ± 2.3	8.6 ± 1.3
Longitudinal equivalent diameter (mm)	11.48 ± 5.0	9.4 ± 2.0	10.3 ± 4.0	9.0 ± 2.5
Volume (mm³)	1596.0 ± 2624.0	630.3 ± 359.3	1247 ± 1925	588.5 ± 377.8
a (mm)	–	4.1 ± 0.7	–	3.9 ± 0.8
b (mm)	–	7.6 ± 1.6	–	7.1 ± 1.5
c (mm)	–	4.4 ± 0.8	–	4.2 ± 0.6

Overview of the mean values of Area and Equivalent diameter for both transverse and longitudinal sections, a, b, and c semiaxes, and lesion volume. The values refer to both variable and fixed time of treatment (VT+FT), and only fixed time groups (FT).

**Figure 6:**
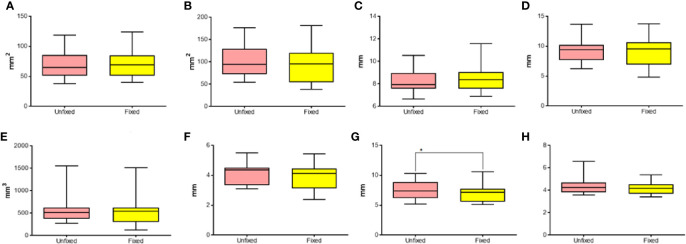
Comparison of different parameters between unfixed and FF thyroids. In details, **(A)** transverse area; **(B)** longitudinal area; **(C)** transverse equivalent diameter; **(D)** longitudinal equivalent diameter; **(E)** volume; **(F)** semi-axis “a”; **(G)** semi-axis “b”; **(H)** semi-axis “c”. *P < 0.05.

The transverse area (**Figure 6A**) and the longitudinal area (**Figure 6B**) were not significantly different between unfixed and FF thyroids (p=0.3392, p=0.3822, respectively). Similarly, the transverse equivalent diameter (**Figure 6C**) and the longitudinal equivalent diameter (**Figure 6D**) were not significantly different between unfixed and FF thyroids (p=0.0808, p=0.3446, respectively).

Similarly, the volume (**Figure 6E**) was not significantly different between unfixed and FF thyroids (p=0.3303). The semiaxis “b” was the only one to be significantly different between the two groups (p=0.0239) (**Figures 6F**–**H**).

During the morphometric analysis of gross thyroids, two halves of the lesion originated from transverse and longitudinal cuts, obtaining two values for the area and the equivalent diameter referred to the same view. The coefficients of variation of these measurements of gross unfixed thyroids were compared with the ones of FF thyroids. No statistically significant differences were detected in the distributions of the coefficients of variation for the transverse area (p=0.0587), for the longitudinal area (p=0.8615), for the transverse equivalent diameter (p=0.3038), and for the longitudinal equivalent diameter (p=0.6556). Generally, the ablated area showed shrunk and darker colloid in follicular tissue, creating an empty space between the follicular wall and the colloid.

**Figure 7:**
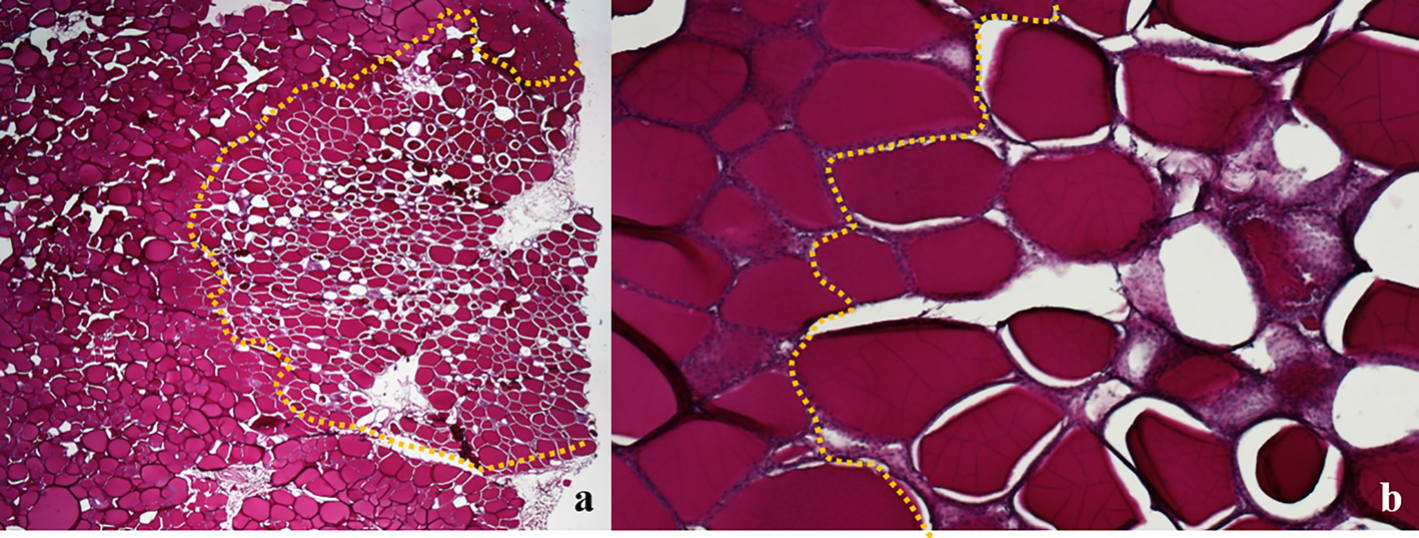
Swine thyroid treated with Wet Tip electrode, 0.9% NaCl solution, variable time of treatment; longitudinal view PAS; the dotted line outlines the ablated areas, showing shrunk and darker colloid in follicular tissue, with empty spaces between the follicular wall and the colloid: **(A)** 20X; **(B)** 100X.

Since the colloid is PAS-positive, the ablation area was easily recognizable in the sections stained with PAS, easily allowing the morphometric analysis (**Figure 7**). No difference was noticed between thyroids treated with IC or WT electrode, apart from larger ablated areas in the latter treatment group.

### Microscopic Morphometric Data

The histological morphometric data are reported in **Table 4**.

**Table 4:** Morphometric data of histological sections of treated thyroids.

Electrode	Solution	Time of treatment	Transverse section	Longitudinal section		a (mm)	b (mm)	c (mm)	Volume (mm³)
			Area T/2 (mm²)	Area (mm²)	Equivalent diameter (mm)				
Internally cooled	–	20-sec fixed time	15.7 ± 5.7	33.1 ± 20.1	4.8 ± 2.0	2.3 ± 1.2	3.8 ± 1.3	3.2 ± 0.8	130.5 ± 97.8
Wet tip	0.9% NaCl	Variable time	99.5 ± 81.3	149.7 ± 119.9	7.9 ± 5.2	4.5 ± 2.3	9.5 ± 5.5	7.2 ± 4.7	1987.0 ± 2407.3
Wet tip	7% NaCl	Variable time	183.2 ± 38.8	543.8 ± 171.2	19.1 ± 4.5	9.3 ± 1.0	18.4 ± 4.8	12.1 ± 2.3	8465.5 ± 1999.4
Wet tip	0.9% NaCl	20-sec fixed time	43.0 ± 18.7	131.7 ± 28.3	8.5 ± 0.9	4.4 ± 0.9	7.9 ± 0.5	5.6 ± 2.0	846.2 ± 430.8
Wet tip	7% NaCl	20-sec fixed time	39.2 ± 16.0	87.2 ± 43.8	6.7 ± 1.7	3.9 ± 1.0	6.7 ± 2.7	4.7 ± 1.6	615.5 ± 530.8
Wet tip	18% NaCl	20-sec fixed time	19.3	74.5	6.9	3.6	8.0	3.4	406.3

Overview of the mean values of Area and Equivalent diameter for both transverse and longitudinal sections, a, b, and c semiaxis, and lesion volume. Only one sample was available for wet tip with 18% NaCl solution.

### Effects of Time of Treatment and Solution on WT Electrode Performances

The measurements of the transverse section were not considered, since the entire view of the lesion was not available due to the trimming procedure.

**Figure 8:**
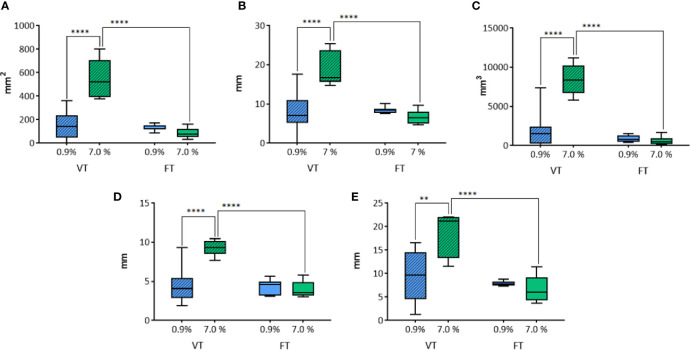
Comparison of the different parameters among the groups treated with WT, with variable and fixed time of treatment, and 0.9% and 7.0% NaCl solutions in histological morphometric analysis. In details, **(A)** longitudinal area; **(B)** longitudinal equivalent diameter; **(C)** volume; **(D)** semi-axis “a”; **(E)** semi-axis “b”. VT, Variable Time treatment; FT, Fixed Time treatment; 0.9%= solution perfused into the tissue at 0.9% NaCl; 7.0%= solution perfused into the tissue at 7.0% NaCl; *P < 0.05; ****P < 0.0001.

The longitudinal area (**Figure 8A**) was influenced by the solution (p=0.0001) and the duration of treatment (p<0.0001). In the VT groups, a significant larger area of ablation was detected when 7.0% NaCl solution was applied, in comparison to 0.9% (p<0.0001). No statistically significant difference was revealed in fixed time groups (p=0.6334). The longitudinal area in the groups treated with 7.0% NaCl solution was significantly larger in the group treated for a variable time in comparison to the fixed time’s one (p<0.0001). No difference was observed between the groups treated with 0.9% NaCl. The longitudinal equivalent diameter (**Figure 8B**) was influenced by the solution (p=0.0018) and the duration of treatment (p=0.0002).

In the groups where the duration of treatment was variable, a significant difference according to the use of 0.9% or 7.0% NaCl solution (p<0.0001) was detected. No significant difference was revealed in fixed time groups (p=0.559). The time of treatment influenced the longitudinal equivalent diameter in the groups treated with 7.0% NaCl solution, where a significant difference between variable and fixed time (p<0.0001) was detected. No difference was found in the groups treated with 0.9% NaCl.

Also the volume (**Figure 8C**) was influenced by the solution (p<0.0001) and the duration of treatment (p<0.0001). In VT groups, a significant difference depending on the use of 0.9% or 7.0% NaCl solution (p<0.0001) was detected. No difference was observed in fixed time groups (p=0.9515). The time of treatment influenced the volume only in the groups treated with 7.0% NaCl solution, where a significant difference between variable and fixed time (p<0.0001) was detected. No difference was found in the groups treated with 0.9% NaCl.

The semi-axis “a” (**Figure 8D**) was influenced by the solution (p=0.0009) and the time of treatment (p<0.0001). In the group where the time of treatment was variable, a significant difference related to the use of 0.9% or 7.0% NaCl solution (p<0.0001) was detected. No significant difference was observed in fixed time groups (p=0.787). The variable time of treatment influenced the semi-axis “a” in the groups treated with 7.0% NaCl solution, revealing a significantly longer “a” semiaxis, in comparison to the homologous treatment at fixed time (p<0.0001). No difference was found in the groups treated with 0.9% NaCl.

The semi-axis “b” (**Figure 8E**) was influenced by the solution (p=0.0165) and the duration of treatment (p=0.0002). In the groups where the time of treatment was variable, a significant difference related to the use of 0.9% or 7.0% NaCl solution (p=0.001) was detected. No significant difference was revealed in fixed time groups (p=0.8144). The time of treatment influenced the semi-axis “b” only in the groups treated with 7.0% NaCl solution, where a significant difference between variable and fixed time (p<0.0001) was detected. No difference was found in the groups treated with 0.9% NaCl.

### Comparison of IC and WT Electrodes Performances

The performances of IC (control group) and WT electrodes (0.9% and 7.0% NaCl) on unfixed thyroids were compared at 20 seconds fixed time of treatment, analyzing microscopic measurements. The transverse section was not considered, since the entire view of the lesion was not available due to the trimming procedure.

**Figure 9:**
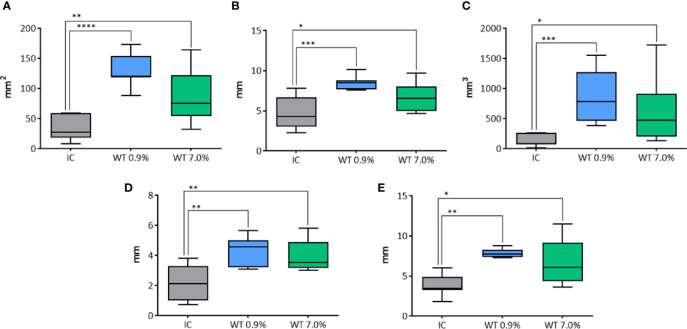
Comparison of different parameters among the groups treated with IC and WT electrodes, and solutions in histological morphometric analysis. In details, **(A)** longitudinal area; **(B)** longitudinal equivalent diameter; **(C)** volume; **(D)** semi-axis “a”; **(E)** semi-axis “b”. IC= Internally cooled tip; WT, Internally cooled wet tip; WT 0.9%-7.0%= solutions at 0.9%-7.0% NaCl perfused into the tissue by the WT electrode; *P < 0.05; **P < 0.01; ***P < 0.001; ****P < 0.0001.

The longitudinal area was affected by the use of either IC or WT electrodes (p<0.0001) at 20 seconds fixed time treatment (**Figure 9A**). In particular, a significant increase of longitudinal area was revealed with WT, in comparison to the control group IC, both using 0.9% NaCl (p<0.0001), and 7.0% NaCl (p=0.0094) solution. The longitudinal equivalent diameter was affected by the use of either IC or WT electrodes (p=0.0014) at 20 seconds fixed time treatment (**Figure 9B**). A significant difference was detected between the control group IC and WT using 0.9% NaCl solution (p=0.0007), and between the control group IC and WT with 7.0% NaCl (p=0.0287). The volume was affected by the use of either IC or WT electrodes (p=0.0002) at 20 seconds fixed time treatment (**Figure 9C**). In particular, a significant increase of the lesion volume was detected in comparison with the control group IC using WT both with 0.9% NaCl solution (p=0.0009), and with 7.0% NaCl (p=0.0243). The semi-axis “a” was affected by the use of either IC or WT electrodes (p=0.0031) at 20 seconds fixed time treatment (**Figure 9D**). In particular, a significant difference was detected between the control group IC and WT using 0.9% NaCl solution (p=0.0025), and between the control group IC and WT with 7.0% NaCl (p=0.0069). Also the semi-axis “b” was affected by the use of either IC or WT electrodes (p=0.0007) at 20 second fixed time treatment (**Figure 9E**). A significant difference was detected between the control group IC and WT using 0.9% NaCl solution (p=0.0015), and between the control group IC and WT with 7.0% NaCl (p=0.0466).

### Comparison Between Macroscopic and Microscopic Morphometric Analysis

The parameters calculated on macroscopic images from unfixed thyroids were compared to the corresponding data obtained from histological images (**Table 5**). The comparison was related to groups where the time of treatment was fixed at 20 seconds.

**Table 5:** Morphometric data of macroscopic images from unfixed thyroid and corresponding histological sections.

Measurement	Gross morphometric analysis; unfixed thyroids	Histological morphometric analysis
Longitudinal area (mm²)	102.9 ± 35.5	83.7 ± 50.0
Longitudinal equivalent diameter (mm)	9.6 ± 1.9	6.7 ± 2.1
Volume (mm³)	634.1 ± 352.0	529.0 ± 478.4
a (mm)	4.3 ± 0.9	3.6 ± 1.3
b (mm)	9.6 ± 1.9	6.7 ± 2.1

Overview of the mean values of Longitudinal Area and Equivalent diameter, lesion volume, a and b semiaxes. The values refer to groups with 20 seconds fixed time of treatment.

**Figure 10:**
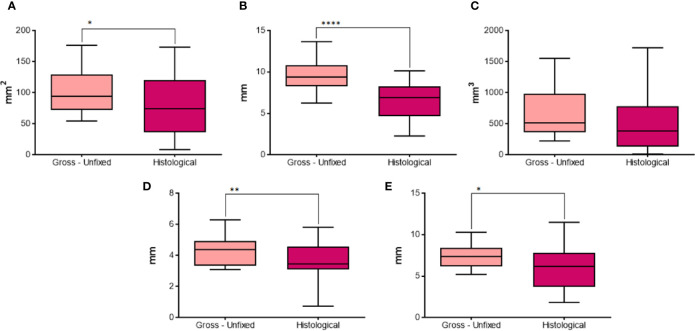
Comparison of different parameters between gross unfixed and histological morphometric analysis in groups with 20 seconds fixed time treatment. In details, **(A)** longitudinal area; **(B)** longitudinal equivalent diameter; **(C)** volume; **(D)** semi-axis “a”; **(E)** semi-axis “b”. *P < 0.05; **P < 0.01; ****P < 0.0001.

The longitudinal area (**Figure 10A**) was significantly larger in pictures from gross unfixed thyroids compared to the corresponding histological images (p=0.0466). The calculated longitudinal equivalent diameter (**Figure 10B**) was significantly longer in gross unfixed pictures compared to histological images (p<0.0001). The calculated volume (**Figure 10C**) was not significantly different between gross unfixed and histological lesions (p=0.1424). The semi-axis “a” (**Figure 10D**) and the semi-axis “b” (**Figure 10E**) were significantly longer in gross unfixed thyroids compared to their histological images (p=0.0046 and p=0.0113, respectively).

## Discussion

Minimally invasive image-guided thermal ablation has become common since the advent of modern imaging (2). Among all, RFA is becoming extremely popular in human medicine. It is the first commercially viable ablation device, it is currently widely available and it is relatively cost effective, compared to other newer devices (24). Moreover, RF is a versatile technique of thermal ablation, used in the treatment of small tumors of multiple organs, but also in cardiac diseases or denervation. The electrodes are usually of small size, and the safety profile is acceptable, especially in the treatment of thyroid nodules. RFA has also a hemostatic effect, helpful in periprocedural bleeding. In addition, the interactions with the immune system, together with its synergy with immunotherapy and conventional therapy, are interesting advantages in tumor treatment (24). Surgery is the primary strategy of therapy for patients with medullary thyroid cancer and differentiated thyroid cancer (DTC). In DTC patients, radioactive iodine is administered after thyroidectomy, but the iodide uptake ability of DTC cancer cells may be lost, with a negative impact on the prognosis (25). Recently, several drugs have been developed, and tyrosine kinase inhibitors play an important role in the molecular pathways of DTC, modulating several pathways involved in angiogenesis, lymphangiogenesis, cell proliferation, local and distant spread of cancer cells (26).

RFA main limitations are the lack of consistency when attempting to obtain larger homogenous tumor ablations, as well as the time needed to deliver sufficient energy with RF ablation (24). The main reasons of limited ablation are related to heat loss and inefficient thermal conduction.

The heat loss is mainly due to the hyperthermia dissipation effect caused by flowing blood or air. Many possible solutions have been developed in the last few years, such as vascular ablation techniques (3) and pharmacological agents (2). The inefficient thermal and electrical conduction is correlated with the intrinsic characteristics of the tissue (i.e. fibrous mass, bone), and by the development of desiccated and carbonized tissue around the electrode. The use of IC electrode may prevent temperatures above 100°C to develop, and the consequent tissue carbonization, leading to this insulating area. However, in this context, also WT electrodes have been developed, which combine the internal cooling system of IC electrodes with the injection of a solution in the tissue through side holes.

Studies on animal livers revealed that WT electrodes have the potential to generate larger, but less reproducible, AZs than IC. The larger size of the AZs may be attributed to the lower impedance resulting from a small amount of spilled saline (20). A study conducted on humans confirmed these results (27). However, no study was conducted on thyroids yet. Hence, the current study compared the performances of IC and WT electrodes on *ex-vivo* swine thyroids. Pigs were the selected species, like in other similar studies (23, 28), since swine thyroid glands have an adequate size and a fine tissue structure resembling that of humans.

In order to have an objective comparison, in this study morphometric analysis was conducted on thyroids, which were unfixed and formalin fixed, and on the corresponding histological sections. Particular attention was paid to the solution used with WT electrodes, and to the duration of treatment.

Thyroid radiofrequency ablation guidelines of the Korean Society of Thyroid Radiology recommended the moving-shot technique as the standard procedure for RFA of benign thyroid nodules (27, 28). However, the moving-shot approach was not considered suitable in this study, intended to perform morphometry and to obtain the more precise measurement and repeatability. In fact, moving-shot technique results are dependent on the number of passages and the tilt angles of the electrode, less replicable than a single passage. The latter allowed a more precise measurement of the parameters obtained by the ablation through the different electrodes and saline solutions.

To determine the effects of saline solution on ablation size, a fixed time procedure was also implemented, stopping energy deliver, independently from the completion of ablation, after 20 seconds, also for perfused electrodes. The procedure was implemented due to the small size of thyroids and following the finding that many of them were completely necrotized with the automatic procedure, thus interfering with a precise measurement of the ablation. The fixed time procedure was intended to better clarify the performances of WT electrodes in terms of ablation size and it is not to be considered for clinic purposes, since the benefit of WT electrodes is that they make larger ablations, but they require longer time. In fact, WT electrodes delay the impedance roll off and surely limiting time to 20 seconds annul the advantage of their use. However, in many cases measurements were not available on variable time treatments with WT electrodes, since the entire pig thyroid was ablated.

During the treatment, the ablation process was visible by sonography as an expanding hyperechoic area into the normal, surrounding tissue.

In unfixed thyroids, the coagulated areas were clearly distinguishable from the normal tissue, with a defined outline in IC treated thyroids, whereas the one obtained with WT electrode appeared to be vanishing. This may be ascribable to the increased thermal and electrical conduction of the tissue caused by the saline solution provided by WT electrode, which let the heat expand more easily and not to be focused in a restricted area, as in IC.

All the measurements, except the “c” semi-axis, carried out with the WT electrode were positively influenced by the use of 7.0% compared to 0.9% NaCl solution in the case of variable time treatments and positively influenced by the duration of treatment. In fact, the variable time treatment achieved significantly larger lesions than the fixed time technique, independently from the solution used (0.9% or 7.0% NaCl). The “c” semi-axis was also significantly longer with 7.0% than 0.9% NaCl solution at variable time of treatment. However, the variable time of treatment achieved higher values of “c” only when 7.0% NaCl was used.

At fixed time of treatment, the transverse area was significantly larger when the thyroid was treated with WT electrode and 0.9% NaCl solution compared to IC electrode and the “c” semi-axis was significantly longer if the WT electrode was used with 0.9% or 18% NaCl solution compared to IC electrode. Therefore, the type of electrode and solution used greatly influenced the measurements obtained on transverse sections. All the other measurements were not significantly different among the groups, even though it could be often noticed a gradual increase of each measurement from IC to WT 18% NaCl solution.

The “a” and “c” semi-axes are considered interchangeable in many studies (23, 29) and this study confirms the literature reports. This means that, even though the use of measurements from transverse section may guarantee more precise results, they are not strictly necessary for comparing performances of different electrodes.

In gross morphometric analysis on FF thyroids, the ablated area was usually better distinguishable than the one of corresponding unfixed thyroids. Usually, the formalin fixation causes a shrinkage effect on tissues. This would mean that the morphometric measurements should have evidently lower values in fixed than unfixed thyroids. When both variable and fixed time groups were included, longitudinal area, longitudinal equivalent diameter and volume were significantly lower in FF than unfixed thyroids. However, the measurements from variable time groups may affect the results, due to their wide variability. In fact, considering only groups with fixed duration of treatment, only the “b” semi-axis was significantly shorter in FF thyroids. Therefore, the effect of formalin fixation on the thermal lesion induced on thyroids seems to be inconstant. It may be influenced by the coagulation and the presence of water.

Moreover, results showed that connective tissue does not influence gross morphometric analysis.

The 18% NaCl solution caused some technical problems during the experiment, such as obstruction of the cooling system of the electrodes. These conflict with the clinical applications.

The coagulated area was clearly recognizable on histological sections, showing follicular tissue with colloid shrinkage and accumulation. However, it shall not be excluded that the parenchyma that is really ablated could be larger than the area histologically detected, due to a delayed damage induced by thermal stress in the following hours and days. In our study, the evaluation was performed on ex vivo tissues obtained soon after slaughtering, and samples were collected immediately after RFA, with no subsequent evaluation planned. The evaluation of a subsequent shrinkage, delayed in time, could require a time lapse collection of thermo ablated tissues, which could be the target of a future study, once established the best performances and conditions for RFA.

Moreover, our study on *ex vivo* tissues did not allow to investigate possible mechanisms of cell death, since vital processes are not active in explanted tissues. Evaluation of apoptosis could be worth of interest in future *in vivo* experiments.

The performances achieved by the WT electrode with the 7.0% NaCl were consistently higher than 0.9%, confirming results of gross morphometric analysis. Moreover, the variable duration of treatment allowed to achieve larger lesions only when the 7.0% NaCl solution was used, partially confirming the results of gross analysis. Further studies should be conducted to better clarify this finding.

WT electrode with 0.9% NaCl solution achieved larger lesions than IC at fixed time of treatment, moreover, WT electrode with 7.0% NaCl solution produced larger ablation than IC. Differently from results on gross morphometric data, even though each measurement appeared to gradually increase its values from IC to WT electrode and 0.9%, 7.0%, and 18% NaCl solutions, only the measurements taken on transverse section revealed a statistical relevance.

Comparing the longitudinal area and equivalent diameter, the volume, and “a” and “b” semi-axis in groups with fixed time duration of treatment, all the measurements on histological sections were significantly lower than on gross unfixed images, except for the volume. However, the thicker half of the lesion was usually selected for trimming the transverse face of the lesion, which makes the “c” semi-axis, and consequently the volume of the rotation solid, probably overestimated.

The reason of lower values in measurements taken on histological analysis may be the sectioning of the specimen. In fact, during the sectioning, the cut margin of the lesion could be broken, even partially, and lost. Another explanation could be the tissue shrinkage caused by the slide processing.

The present study showed that the duration of treatment definitely influences the performance of the WT electrode. Therefore, the perfusion with 7.0% NaCl solution does increase the electrical conductivity of the tissue, resulting in larger ablated areas, compared to the use of 0.9% NaCl solution, but the effect is evident only when the electrode works until the generator spontaneously turns off (as in the variable time) and not visible with short duration of 20 seconds. Moreover, the macroscopic analysis revealed that fixed time treatments usually generate smaller lesions compared to variable time treatments, as opposed to the histological analysis, underlining that this is relevant only when 7.0% NaCl solution is used. However, values of the measurements on thyroids treated with variable time and 0.9% NaCl solution are more different than expected and hence probably affected by some errors. More trials should be done to better clarify this finding.

The performances of WT electrode seem higher than the one obtained with IC at fixed time of 20 seconds. In fact, the histological analysis revealed a clear difference between IC and WT 0.9% NaCl, and a lower difference when 7.0% NaCl solution was used. This scenario is similar in gross thyroid analysis, since 0.9% NaCl seems to have a better performance than IC electrode and WT electrode with 7.0% NaCl at fixed time, although the difference is not statistically significant.

This fact may be attributable to two causes. The former is the lower statistical power of the analysis conducted on gross thyroids, where the comparison involved four groups (IC, WT 0.9% NaCl, WT 7.0% NaCl, and WT 18.0% NaCl), instead of the three groups of histology (where WT 18.0% NaCl was not included). The latter is the usually more precise outline of the ablated area boundary in the case of histology, which is less subjected to subjectivity, compared to gross analysis. The results indicate that the performances of different electrodes evaluated on gross unfixed lesions and on microphotographs agree.

In conclusion, the histological evaluation seems to be the best approach for morphometric analysis of RF ablated area on *ex-vivo* thyroids, showing a high potential. The WT electrode performances on thyroid are superior compared to IC. A similar finding was already reported for liver treatments (20, 22, 27).

Therefore, WT electrodes demonstrated to give larger AZs, if they are correctly used at their best, i.e. at a variable time. The advantage could be transposed in the clinical procedures, allowing treatments with a fewer number of shots in large thyroidal nodules. Moreover, WT electrode performances seem to depend on the solution used. In particular, the injection of 7.0% NaCl solution may be advantageous, because it achieves larger ablation in variable time procedures, such as the one usually adopted in clinical practice in the treatment of thyroid nodule as well as diseases in other organs in human medicine.

Percutaneous energy-based techniques have an expanding role, especially in the treatment of neoplasms (2). Hopefully, the current work will contribute to broaden the clinical indications of RFA in both human and veterinary medicine, where the ablation size has limited its use so far.

## Data Availability Statement

The raw data supporting the conclusions of this article will be made available by the authors, without undue reservation.

## Ethics Statement

Ethical review and approval was not required in accordance with national guidelines and local legislation for the animal study because thyroids were collected from regularly slaughtered swine, during meat production procedures. The thyroids were collected at the slaughterhouse among discarded organs, not destined for human consumption. This type of sampling doesn’t require any ethical approval.

## Author Contributions

PP: study design, statistical analysis, paper drafting. ES: experimental procedures. MB: statistical analysis, paper drafting MM: echographic procedures. LN: Morphometric analysis. SG: echographic procedures. RG: RFA procedures. EB: morhometric analysis, paper revision. FS: study design, paper drafting, coordination of the group. All authors contributed to the article and approved the submitted version.

## Conflict of Interest

The authors declare that the research was conducted in the absence of any commercial or financial relationships that could be construed as a potential conflict of interest.

The reviewer [LP] declared a shared affiliation with several of the authors, [PP, MB, LN, SG, RG, EB, FES], to the handling editor at time of review.

## Abbreviations

AZ, Ablation zone; CT, computed tomography; DTC, differentiated thyroid cancer; MR, Magnetic Resonance; RF, Radiofrequency; RFA, Radiofrequency ablation; US, Ultrasound; IC, Internally cooled and non-perfused electrodes; WT, Internally cooled and perfused (wet tip) electrodes; FF, Formalin fixed; VT, Variable Time treatment; FT, Fixed Time treatment.
